# Towards Highly Efficient Polymer Fiber Laser Sources for Integrated Photonic Sensors

**DOI:** 10.3390/s20154086

**Published:** 2020-07-22

**Authors:** Simon Spelthann, Stefanie Unland, Jonas Thiem, Florian Jakobs, Jana Kielhorn, Pen Yiao Ang, Hans-Hermann Johannes, Dietmar Kracht, Joerg Neumann, Axel Ruehl, Wolfgang Kowalsky, Detlev Ristau

**Affiliations:** 1 Institute of Quantum Optics, Leibniz University Hannover, 30167 Hannover, Germany; spelthann@iqo.uni-hannover.de (S.S.); thiem@iqo.uni-hannover.de (J.T.); d.ristau@lzh.de (D.R.); 2 Laser Zentrum Hannover e.V., 30419 Hannover, Germany; s.unland@lzh.de (S.U.); d.kracht@lzh.de (D.K.); j.neumann@lzh.de (J.N.); 3 Institut für Hochfrequenztechnik, TU Braunschweig, 38106 Braunschweig, Germany; florian.jakobs@ihf.tu-bs.de (F.J.); jana.kielhorn@ihf.tu-bs.de (J.K.); pen.yiao.ang@ihf.tu-bs.de (P.Y.A.); h2.johannes@ihf.tu-bs.de (H.-H.J.); wolfgang.kowalsky@ihf.tu-bs.de (W.K.); 4 Academic Alliance Braunschweig - Hannover QUANOMET, 30167 Hannover, Germany; 5 Cluster of Excellence PhoenixD, 30167 Hannover, Germany

**Keywords:** polymer fiber laser, polymer fiber amplifier, integrated photonics, Rhodamine B, fiber optics

## Abstract

Lab-on-a-Chip (LoC) devices combining microfluidic analyte provision with integrated optical analysis are highly desirable for several applications in biological or medical sciences. While the microfluidic approach is already broadly addressed, some work needs to be done regarding the integrated optics, especially provision of highly integrable laser sources. Polymer optical fiber (POF) lasers represent an alignment-free, rugged, and flexible technology platform. Additionally, POFs are intrinsically compatible to polymer microfluidic devices. Home-made Rhodamine B (RB)-doped POFs were characterized with experimental and numerical parameter studies on their lasing potential. High output energies of 1.65 mJ, high slope efficiencies of 56%, and 50%-lifetimes of ≥900 k shots were extracted from RB:POFs. Furthermore, RB:POFs show broad spectral tunability over several tens of nanometers. A route to optimize polymer fiber lasers is revealed, providing functionality for a broad range of LoC devices. Spectral tunability, high efficiencies, and output energies enable a broad field of LoC applications.

## 1. Introduction

Sensors for biological or medical sciences are often bulky and complex, especially optical analysis techniques, which usually consist of several distinct devices, like lasers, optical components, and the analysis platform. As a consequence, such devices are not only complex to operate and maintain but also expensive. However, for spectroscopic approaches, e.g., in point-of-care testing, it is highly desirable to provide cheap, small, and easy to use and fabricate devices.

These requirements have been approached by Lab-on-a-Chip (LoC) devices, e.g., miniaturized gas chromatography systems, holding promising potential for the rapid analysis on a compact and fully integrable platform [1,2,3]. Combining integrated photonics, such as laser systems, with existing LoC approaches is mandatory to fulfill the requirements for modern optical analysis techniques [2,4]. Such LoC devices seem to be in the scope as 3D printers have become more and more sophisticated and affordable [5,6]. Polymers are the easiest to handle via 3D printing and thus constitute one of the most promising laser platforms for LoC devices. Semiconductor light sources, e.g., laser diodes (LD) or light emitting diodes (LED), indeed combine a compact design with tailored emission properties and all-electrical control, but the integration into a polymer platform is difficult and usually done by means of assembly. One batch producible LoC devices would lead to a higher cost effectiveness requiring polymerizable light sources. Organic LEDs were combined with organic photodiodes on glass substrates and then attached to a microfluidic channel for fluorescent sensing [7]. Recently, electrically pumped organic LDs were reported for the first time emitting an optical power of 0.5 mW [8]. Although this approach seems to be promising, the high attenuation in polymers of up to several dB/cm requires higher power levels.

In this paper, we address the needs for laser active gain materials capable of producing high output energies while being easily integrable into a polymer platform: polymer fiber lasers. They provide a rugged and flexible technology platform which enables simple, compact, and alignment-free laser setups. Fiber lasers based on polymers meet the requirement for compatibility with microfluidic platforms, on the one hand, and 3D printing processes, on the other hand. Polymer fiber lasers thus constitute an excellent substitute light source for polymer-based LoC devices.

Laser activity in polymer optical fibers (POF) are mostly realized by doping the fiber core with laser dyes. Highly efficient lasers can be set up, performing on slope efficiencies up to 43% and providing high output energies of 640μJ [9]. Large gain values of up to 27 dB can be exploited in fiber amplifiers [10], but degradation of the laser dyes via photobleaching and thermal load during the pumping process are general weak points, resulting in 50%-lifetimes of 200 k pulses at repetition rates of 10 Hz [11]. Hence, the dye molecules can be damaged when treated thermally. Regarding 3D printing processing polymers above $200{\;}^{{}^{\circ}}$C, the efficiency of a laser will be reduced when integrating the active material in a photonic device. To nevertheless provide sufficient optical output energies, the performance of RB:POF lasers has to be optimized.

We present experimental and numerical studies on the optimization of POF lasers using polymethyl methacrylate (PMMA) doped with Rhodamine B (RB). The lasing characteristics, such as wavelength and slope efficiency, were investigated for laser output couplers (OC) with different degrees of reflection. Laser experiments were compared to numerical simulations.

## 2. Manufacturing and Characteristics

The PMMA preforms were prepared using radical polymerization in bulk with 0.03 mol% lauroyl peroxide as initiator and 0.1 mol% n-butyl-mercaptan as chain transfer agent. RB was mixed with the initiator and the chain transfer agent in nitrogen-saturated methyl methacrylate. The solution was polymerized and the final RB:PMMA preforms were drawn in a self-made drawing tower to fiber cores. The drawn fibers had a length between 20 and 30 m, depending on the particular preform length. In a second step, the fiber is coated with acrylate and drawn through a nozzle. The cladding was cured with UV light afterwards. The diameter of the resulting core ranged from 920–1000 μm, whereas the total diameter was 1150μm. These dimensions are oriented by standard POFs which have a core diameter of (980±60)μm and a total diameter of (1000±60)μm. The diameter of our fibers exceeds the value of a standard POFs to account for variations of the fiber cores and assure a certain cladding thickness. More details on the preparation and characterization process for dye-doped POF can be found in References [12,13]. Fibers with different doping concentrations of 1 ppm, 5 ppm, and 10 ppm were manufactured and used for the experiments. Note that these values refer to the amount of RB which was added to the synthesis. The exact value may slightly deviate due to the thermal treatment during the polymerization and the fiber drawing process.

Absorption and emission spectra and fluorescence lifetime, as well as the attenuation of the fiber, were measured to deduce experimental parameters but also for use as input parameters for numerical investigations concerning the RB:POF lasers. The attenuation was determined using a 4–5 m long fiber and cutting it back about 10 times by increments of 0.3 m.

**Figure 1:**
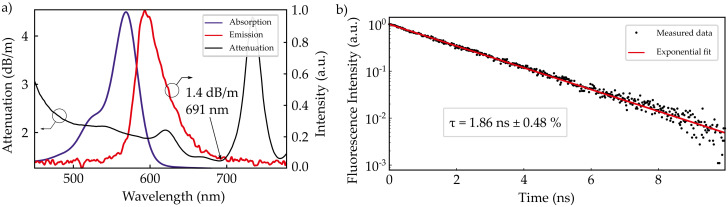
Spectral characteristics of the used Rhodamine B:polymer optical fibers (RB:POFs). (**a**) Absorption and Emission spectra together with the spectral attenuation. (**b**) Fluorescence lifetime of RB-doped polymethyl methacrylate (PMMA) used for fiber manufacturing.

The absorption spectrum was measured for RB-doped PMMA preforms with a doping concentration of 1 ppm with the maximum located at 557 nm as depicted in Figure 1a. The absorption maxima of 5 ppm and 10 ppm differ by Δλ≤ 2 nm, consistent with previous reports, e.g., Ref. [14]. The emission spectra are red shifted with respect to the absorption spectra due to the Stokes shift. Depending on the doping concentration, the emission maxima range between 595 nm and 618 nm. The absorption and emission spectra overlap, which causes reabsorption, resulting in a greater Stokes shift for high doping concentrations [15]. We further manufactured a passive POF in the same way as described above. Cut back experiments were carried out with a supercontinuum light source to determine the spectral dependence of the fiber attenuation, depicted in Figure 1a. Infrared attenuation is not shown in the figure because the attenuation exceeds values which are reasonable for laser applications. The attenuation minimum of 1.4 dB/m is located at 691 nm. Measurements of the fluorescence decay with a streak camera resulted in a fluorescence lifetime of 1.86 ns, as shown in Figure 1b. Such low upper state lifetimes result in high lasing thresholds, thus implying high pump energies, e.g., available from q-switch pulsed laser systems. The fluorescence lifetime of RB in ethanol is about 2.7 ns [16], thus deviating by only 30 % from its typical case of application.

## 3. Numerical Model

We used a system of rate equations to verify our experimental results numerically. These rate equations were discretised for spatial dimension *z* and the wavelength λ of the propagating light. We assume a four level system with a fast radiative decay between levels three and two, as well as levels one and zero. As a consequence, steady-state operation of our RB:POF laser can be modeled with a system of two energy levels with population numbers $N_{2}$ of the upper laser level and $N_{1}$ of the lower laser level, where the derivatives with respect to time d/dt are set to zero [17]:

(1) $$\frac{dN_{2}}{dt}=-\frac{N_{2}}{\tau }+\left(\frac{\sigma_{abs}\left(\lambda \right)P^{\pm }\left(z,\lambda \right)}{h\nu A}N_{1}-\frac{\sigma_{em}\left(\lambda \right)P^{\pm }\left(z,\lambda \right)}{h\nu A}N_{2}\right),$$

(2) $$\frac{dN_{1}}{dt}=-\frac{\sigma_{abs}\left(\lambda \right)P^{\pm }\left(z,\lambda \right)}{h\nu A}N_{1}+\frac{\sigma_{em}\left(\lambda \right)P^{\pm }\left(z,\lambda \right)}{h\nu A}N_{2},$$

(3) $$N_{total}=N_{1}+N_{2}.$$

In these equations, τ is the fluorescence lifetime of the upper laser level 2, and $\sigma_{abs}$ and $\sigma_{em}$ are the cross sections for absorption and emission, respectively. $N_{1}$ and $N_{2}$ constitute the population of the corresponding energy levels, which is determined by the concentration density of the dye molecules, and *A* is the cross section of the fiber core. Forwards (+) and backwards (−) propagation are superscripted in the equations. All parameters were set to SI units. The propagation of the laser power can be described by

(4) $$\frac{dP^{\pm }}{\pm dz}=\sigma_{em}\left(\lambda \right)P{\left(z,\lambda \right)}^{\pm }N_{2}-\sigma_{abs}\left(\lambda \right)P{\left(z,\lambda \right)}^{\pm }N_{1}+\frac{N_{2}hc}{\tau \lambda }\epsilon_{em,sp}\left(\lambda \right)\beta A,$$

where β is the amount of fluorescence that is guided within the fiber which depends on the numerical aperture of the fiber. The efficiency of spontaneous emission $\epsilon_{em,sp}$ is calculated by normalizing the corresponding cross section $\sigma_{em}$ to an integral value of 1. We used the shooting method [18] to numerically solve the equations for the boundary conditions of a fiber laser system with reflectivities in the laser resonator of $R_{1}$ and $R_{2}$.

(5) $$P^{-}\left(z,\lambda \right)=P^{+}\left(l,\lambda \right)R_{2}\left(\lambda \right),$$

with *l* the length of a particular fiber and

(6) $$P^{+}\left(0,\lambda \right)=P^{-}\left(0,\lambda \right)R_{1}\left(\lambda \right).$$

The shooting algorithm calculates the propagating powers $P^{+}$ and $P^{-}$ and iteratively minimizes the difference between $P^{+}\left(z\right)\cdot R2$ and $P^{-}\left(z\right)$ by changing the input power levels at z=0. The pump rate has to be modified as we used a transversal pump scheme. Rate equations for longitudinally pumped fiber lasers are mostly implemented with the pump rate decreasing exponentially along the fiber. In our case, this decrease occurs along the fiber cross section, which is why the pump rate is set constant along the fiber. The pump power is constant within each spatial increment dz over the length of the fiber. Transversal pump light distribution is averaged through the fiber cross section. The extracted laser power is calculated from the boundary conditions, where absorption inside the mirror is neglected.

(7) $$P_{Lasing}\left(\lambda \right)=P^{+}\left(z,\lambda \right)\left(1-R_{2}\left(\lambda \right)\right).$$

The spectroscopic data and fiber properties presented in Section 2 were used as input parameters to solve Equations (1)–(7).

## 4. RB:POF Laser

Rhodamine B (RB)-doped POF lasers have already been investigated by Kuriki et al. [9]. Although this work was done 20 years ago, it still denotes the best values on the performance of dye-dopedPOF lasers reported so far. Kuriki et al. used gradient index (GI) POFs with diameters of 0.6 mm and 1 mm, respectively. These GI-POFs were doped with 1 ppm of different organic dyes, inter alia RB. The POFs with a particular length of 5 cm were pumped transversally using millijoule pulses at 532 nm, and the polished facet formed the resonator together with a butt coupled dielectric mirror.

**Figure 2:**
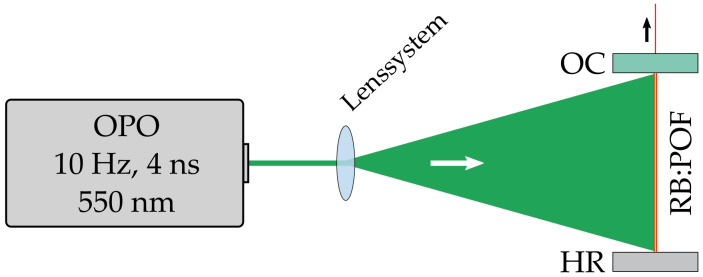
Schematic of the RB:POF laser transversally pumped with an optical parametric oscillator (OPO). The resonator mirrors are both butt coupled to the fiber facets. OC: output coupler; HR: high reflective mirror.

In our investigation, we also used a transversal pumping scheme as sketched in Figure 2 to avoid bleaching. Photo bleaching may occur due to triplet-triplet transitions within a dye molecule, but high pump energies can also destroy the dye molecules thermally [19,20]. In preliminary experiments on different pumping schemes in RB:POF amplifiers, the bleaching rate was almost 60-fold lower for the transversal geometry compared to the traditional longitudinal pumping.

As a pump source, we used an optical parametric oscillator (OPO), itself pumped by the third harmonic of a q-switched Nd:YAG laser providing pulse energies up to 70 mJ and pulse durations of 4 ns. Kuriki et al. pumped their POF lasers at 532 nm where pump absorption is estimated to be 50% lower than at absorption maximum. For optimum pump absorption in our material, the OPO was tuned to 550 nm. This deviation from the wavelength of maximum absorption, stated in Section 2, was necessary due to technical reasons. Although the pump wavelength set in our experiments deviates by 7 nm, pump absorption is estimated to differ by only 1.5%, which was considered to be acceptable.

Our laser consisted of a linear cavity, including a 6 cm gain fiber. In order to prepare the RB:POFs for the experiments, they were cut into the particular length using a POF-cutter. To release internal stress originating from the fiber drawing process the fibers were tempered in an oven at $95{\;}^{{}^{\circ}}C$ for 10 min. Afterwards, the facets were polished in a three-step procedure to obtain a clean and smooth facet surface. The fiber was mounted in an SMA plug connected to a polishing puck. Polishing films with grain sizes of 3μm, 1μm, and 0.3μm were used to polish the fibers on a glass plate.

A silver coated high reflective mirror and a variable dielectric output coupling mirror (OC) both butt-coupled to the fiber endfaces formed the resonator. Note, that the wavelength dependency of the used OC reflectivity results in small deviations of the reflectivity for the differently doped fibers. Different doping concentrations, as well as the OC reflectivity, were investigated in a parameter study to determine optimum parameters towards high efficiencies and output energies.

### 4.1. Optimization of the Laser Efficiency

**Figure 3:**
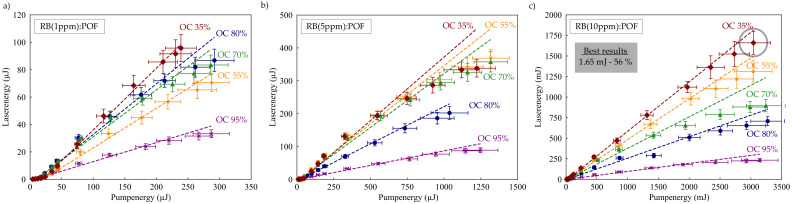
Characteristic curves of the lasers for doping concentrations of (**a**) 1 ppm, (**b**) 5 ppm, and (**c**) 10 ppm. Saturation effects were observed, especially for the 5 ppm doped fiber laser.

Detailed characterization of the laser performance was performed depending on the doping concentration and resonator quality to identify configurations for highly efficient lasing. Output versus pump characteristics of the lasers were recorded and are depicted in Figure 3. Accurate values of the absorbed pump power were obtained experimentally by measuring the transversally transmitted pump energy through an aperture. The slope efficiencies were obtained from a linear fit. In general, laser output energy increases with decreasing OC reflectivity as high amplifications can be exploited from dye-doped POFs. The laser characteristics for the 1 ppm deviate from this observation which might be due to nonuniform distribution of RB molecules within our preforms. Saturation effects were observed for doping concentrations >5 ppm. Increasing doping concentrations only leads to increasing output energies as long as reabsorption and bleaching play a minor role.

We observed a maximum pulse energy of 1.65 mJ by using a 10 ppm POF, together with a 35% output coupler. Taking into account the pulse durations of about 2 ns measured with a fast photodiode, this corresponds to a peak power of 825 kW and constitutes an almost three-fold improvement to the RB:POF laser presented by Kuriki et al. [9]. In this configuration, the slope efficiency of 56% was the maximum value in the parameter range used in this study. This constitutes an improvement of more than 10 percentage points to the RB:POF laser of Reference [9]. To the best of our knowledge, our 10 ppm doped RB:POF laser with an OC reflectivity of 35% thus performed on the highest slope efficiency and provided the highest output energy. Compared to the results from Kuriki et al., we ascribe these improvements to the more suitable pump wavelength and higher resonator quality. Furthermore, we included the doping concentration to our investigations. Not using GI-POFs but normal POFs may also have had an positive influence on the performance of our lasers. Indeed, the fibers act like a cylindrical lens to the pump light due to their shape, leading to an imhomogenous pump distribution within the fiber. This effect is assumed to be stronger in GI-POFs, which Kurike et al. used.

**Figure 4:**
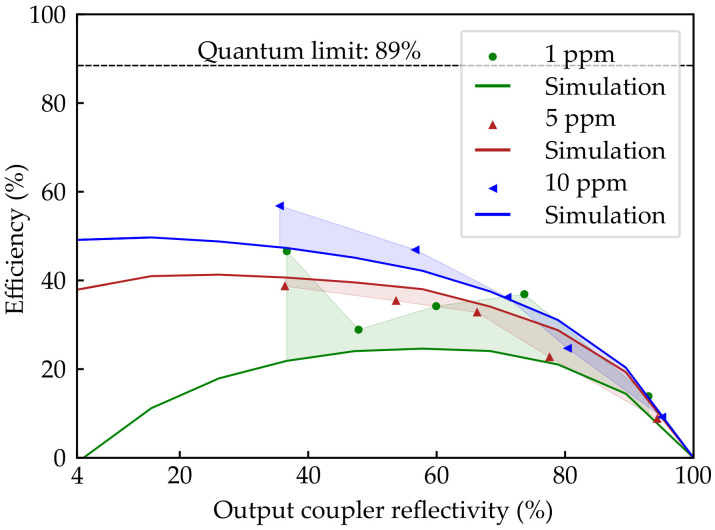
Comparison of the laser efficiencies of all configurations obtained by numerical (solid lines) and experimental results (markers).

As can be seen from Figure 4, where the lasing efficiencies of all configurations are plotted, our numerical model was able to fully support the experimental data of the 5 ppm and 10 ppm doped lasers (Figure 4). Somewhat higher deviations between numerical and experimental results were obtained for the 1 ppm doped fiber. We assume an experimental uncertainty due to a nonuniform distribution of RB molecules in the RB:POF. To ensure that the results do not depend on a particular piece of fiber, the experimental results were confirmed with different pieces of the fiber. Understanding this behavior of the RB(1 ppm):POF laser needs further investigation.

### 4.2. Long-Term Stability of the Laser Operation

Bleaching is the well-known degradation mechanism which reduces the laser output energy of dye-doped laser materials. Long-term measurement of the output energy was performed to characterize this degradation mechanism in our laser systems.

**Figure 5:**
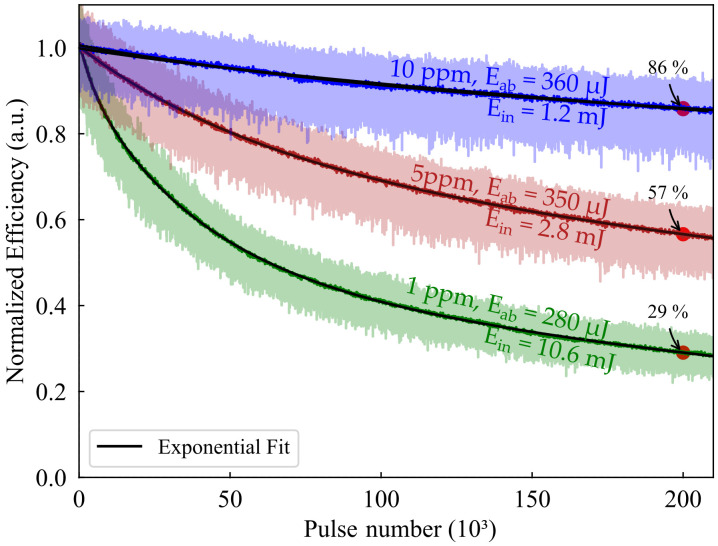
Long-term performance of the RB:POF laser with 1, 5, and 10 ppm doping concentration. The pump energy was scaled towards comparable absorption within each fiber. The measurements are corrected for pump energy fluctuations. Exponential functions were fitted to the data revealing the 50%-lifetimes.

The results are shown in Figure 5. The reflectivity of the OC was set to 70%, and the pump wavelength was tuned to 550 nm. The efficiency of lasers with doping concentrations of 1 ppm, 5 ppm, and 10 ppm were recorded for 200 k shots at 10 Hz, corresponding to 5 h and 33 min with a photodiode and an analog/digital IO device connected to a computer via USB. The incident pump energy differed as the absorbed pump energy scales with the doping concentration, but all fibers had to absorb the same amount of energy for better comparability. The normalized laser efficiency was calculated for better comparability and to prevent falsification due to pump fluctuations.

The output energy of all lasers decreased significantly within the 200 k shots. The normalized efficiency dropped to 86%, 57%, 29% for the 10 ppm, 5 ppm, and 1 ppm doped fiber, respectively. Fitting a mono-exponential function to the measured curves revealed the 50%-lifetime of the fiber lasers to be 905 k, 280 k, and 63 k shots for the 10 ppm, 5 ppm and 1 ppm doped fiber, respectively. Compared with the value of 200 k reported by Kuriki et al. [11], this constitutes a 4.5-fold improvement of the 50%-lifetime. Note that optimization towards long-term stability could presumably lead to even higher 50%-lifetimes.

### 4.3. Spectral Analysis

**Figure 6:**
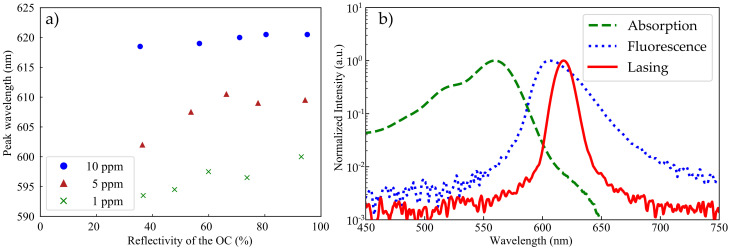
(**a**) Representative absorption, fluorescence, and laser spectrum highlight red shift of the emission wavelength during laser operation. (**b**) Peak wavelength for different resonator configurations. The tuning range is approximately 28 nm for the range of doping concentrations considered and 9 nm within one doping concentration when changing the reflectivity of the output coupler.

The output wavelength of the lasers changed depending on the doping concentration and the OC reflectivity, as it can be deduced from the plot in Figure 6a. The wavelength shifted to longer wavelength for increasing doping concentrations resulting in a total wavelength shift of Δλ≈ 28 nm. For decreasing OC reflectivities, it shifted to shorter wavelength resulting in a wavelength shift of Δλ≈ 9 nm within one doping concentration. The underlying mechanism is enabled by the spectral overlap of absorption and emission spectra. The laser wavelengths are thus spectrally shifted in comparison with spontaneous emission, as can be seen exemplarily in Figure 6b. Fibers with high doping concentrations show a higher number of RB molecules; thus, reabsorption by an RB molecule is more likely than in fibers with low doping concentrations. Analogously, a high OC reflectivity leads to more round-trips of a photon within the resonator, resulting in a high probability for reabsorption. The reabsorbed photons can be re-emitted, which leads to the observed red shifts [21].

These results show one opportunity to tune the wavelength of our RB:POF lasers that can be used to extend the functionality and field of applications [22,23,24].

### 4.4. Numerical Results on the Wavelength Tunability

**Figure 7:**
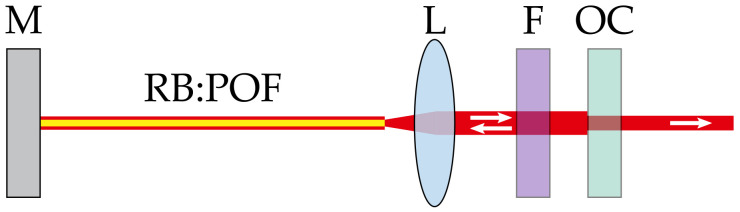
Schematic setup assumed for the numerical investigations on the tunability of a RB:POF laser. M: mirror; L: lens; F: spectral filter; OC: output coupler.

As the tuning range observed with different resonator configuration was still much smaller than the fluorescence bandwidth, we applied our rate equation model to simulate the potential wavelength tunability of our RB:POFs (c.f. Section 3). In order to numerically treat this approach, a wavelength dependent output coupling loss was implemented, representing the combination of a spectral filter with a bandwidth of Δλ=5 nm and output coupling mirror, as sketched in Figure 7. Further input parameters were chosen according to the experimental values which are described in Section 2 and Section 4.

**Figure 8:**
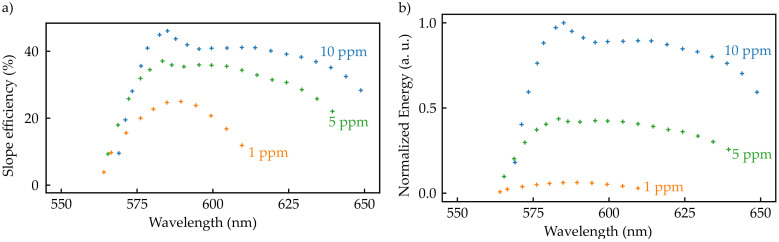
(**a**) Simulated slope efficiency for a tunable polymer fiber laser consisting of our RB:POF. Efficiencies of more than 40% are possible, as well as a tuning range of more than 80 nm. (**b**) Corresponding output energy of such tunable RB:POF laser. Highest output energies are expectable for 10 ppm doped fibers.

The simulations yielded a tuning range of several 10 nm, as can be seen from Figure 8, where the spectral dependence of the slope efficiency, as well as the normalized output energy, is shown. The slope efficiency and output energy show a maximum for 5 ppm and 10 ppm around 580 nm. This may be caused by the local minimum in the spectral attenuation in interplay with the specific spectral location of the emission (c.f. Figure 1). The lasers with all three doping concentrations show high slope efficiencies. RB:POFs doped with 10 ppm can perform with a slope efficiency of more than 40% according to our numerical results. Even a 1 ppm doped RB:POF laser yields a slope efficiency of more than 20%. The tuning range of the lasers broadens with increasing doping concentrations from >40 nm for 1 ppm to >80 nm for 10 ppm due to higher gain for higher doping concentrations.

Exemplary, the RB(1 ppm):POF laser yields only about 10% of output energy compared to a RB(10 ppm):POF laser. While the slope efficiencies for lasers with all three doping concentrations are satisfactory, output energies scale sensitively with the doping concentration in our simulations which is in accordance with experiments (c.f. Section 4.1). To realize such a tunable laser is possible in principle and will be addressed in the future to provide extension of the functionality and field of applications.

## 5. Conclusions

The route to high efficient dye-doped POF lasers was presented in this paper and includes optimization of the pump geometry, pump wavelength, resonator quality, doping concentration, and accompanying numerical treatment. We presented polymer fiber lasers doped with RB, which provide output energies up to 1.65 mJ, high slope efficiencies up to 56%, and 50%-lifetimes of more than 900 k shots. These characteristics constitute a significant optimization compared to Rhodamine-doped POF lasers reported previously [9,11,25,26]. Additionally, coarse wavelength tunabilty of 28 nm can be achieved by varying the doping concentration and fine tunabilty within 9 nm is enabled through change of the resonator quality. The numerical modeling of the RB:POF lasers yielded accurate results compared to the experiments. Furthermore, simulations predict a broad spectral tuning range >80 nm including slope efficiencies of >40% by using a wavelength tunable RB:POF laser setup. Setting up such tunable RB:POF laser is aimed at in the future.

In the future, a work-around strategy is needed to replace the complex and expensive pump laser. The starting point for such further optimization might be the energy storage capacity, which is described through the fluorescence lifetime of the laser dye. In another current work, we address this by using upconversion nanocrystals as active dopand. Prospectively, organic laser active media with cheaper addressable pump transitions, such as electrically pumped organic laser diodes, will be of great interest to investigations on integrated photonic LoC devices. Further attempts to optimize the performance of polymer fiber lasers may be addressed to the attenuation of the material, as well as the stability and fluorescence lifetime of the incorporated dye. We aim to approach a lower attenuation of the material by using deuterated polymers. We also plan to achieve a higher integration grade by sputtering resonator mirrors to the fiber facets. Polymer fiber lasers and amplifiers can be applied to provide wavelength tunable, high efficiency, and energy delivering light sources at reasonable financial effort. The polymer basis of such sources leads to suitability and high integrate-ability for LoC-devices.
